# The Relationship between Binge Drinking and Metabolic Syndrome Components amongst Young Adults Aged 21 to 31 Years: Ellisras Longitudinal Study

**DOI:** 10.3390/ijerph17207484

**Published:** 2020-10-15

**Authors:** Kotsedi Daniel Monyeki, Hlengani James Siweya, Han C. G. Kemper, Andre P. Kengne, Geofrey Musinguzi, Mbelegem Rosina Nkwana, Tebogo Mothiba, Tumiso Malatji, Shisana M.-A. Baloyi, Rambelani Malema, Lloyd Leach, Moloko Matshipi, Ramakgahlela Betty Sebati, Mohlago Ablonia Seloka, Eliot Sibuyi, Suzan Mafoloa Monyeki

**Affiliations:** 1 Department of Physiology and Environmental Health, University of Limpopo, Sovenga 0727, South Africa; Rosina.nkwana@ul.ac.za (M.R.N.); moloko.matshipi@ul.ac.za (M.M.); bettysebati800@gmail.com (R.B.S.); mohlago9592@gmail.com (M.A.S.); eliot.sibuyi@ul.ac.za (E.S.); suzan.monyeki65@gmail.com (S.M.M.); 2 Executive Dean Faculty of Science and Agriculture, University of Limpopo, Sovenga 0727, South Africa; hlengani.siweya@ul.ac.za; 3 Amsterdam Public Health Research Institute, Amsterdam UMC, 1218 HD Amsterdam, The Netherlands; hancgkemper@upcmail.nl; 4 South African Medical Research Council, Francie van Zijl Drive, Parow Valley, Cape Town 7505, South Africa; Andre.Kengne@mrc.ac.za; 5 Department of Disease Control and Environmental Health, School of Public Health, Makerere University, Kampala 7072, Uganda; mgeof@musph.ac.ug; 6 Department of Primary and Interdisciplinary Care, University of Antwerp, 2610 Antwerpen, Belgium; 7 Faculty of Health Science, University of Limpopo, Sovenga 0727, South Africa; tebogo.mothiba@ul.ac.za; 8 Department of Health, Polokwane Provincial Hospital, Polokwane 0700, South Africa; tumimalatji79@gmail.com; 9 Obstetrics and Gynecology, Faculty of Health Sciences, Posbus 339, Bloemfontein 9300, South Africa; BaloyiSM@ufs.ac.za; 10 Department of Psychology, University of Limpopo, Polokwane 0700, South Africa; nancy.malema@ul.ac.za; 11 Department of Sport, Recreation and Exercise Science, University of the Western Cape, Private Bag X17, Bellville, Cape Town 7535, South Africa; lleach@uwc.ac.za

**Keywords:** binge drinking, diabetes, hypertension, overweight, young adult, metabolic syndrome

## Abstract

Background: Evidence is lacking on the effects of binge alcohol consumption on metabolic syndrome in the rural South African population. The purpose of this study was to investigate the association between binge drinking and components of metabolic syndrome (MetS) amongst Ellisras rural young adults aged 21 to 31 years who are part of the Ellisras Longitudinal Study. Methods: Logistic regression analysis was applied to a total of 624 participants (306 males and 318 females) aged 21 to 31 years who took part in the Ellisras Longitudinal Study (ELS). The model was adjusted for covariates, including smoking, age, and gender. Binge alcohol consumption was assessed using a standardised questionnaire that was validated for the Ellisras rural community. A standardised method of determining the components MetS was used after fasting blood samples were collected from all the participants. Results: Binge drinking remained significantly associated with low levels of high-density lipoprotein cholesterol (HDL-C) (OR = 2.64, 95% CI = 1.23–5.65), after being adjusted for smoking, age, and gender. Other MetS components were not predicted. Instead, gender remained significantly associated with all MetS components, except triglycerides, at multivariate analysis. Age retained significance at multivariate analysis with waist girth (OR = 2.13, 95% CI = 1.37–3.34), triglycerides (OR = 2.30, 95% CI = 1.05–5.02), and the MetS composite (OR = 1.65, 95% CI = 1.12–2.41). Conclusion: Binge drinking was significantly associated with lower levels of HDL-C. Future studies should investigate the relationship between alcohol abuse and the components of incident MetS in this population.

## 1. Introduction

Metabolic syndrome (MetS) is characterised by a constellation of metabolic risk factors, including central adiposity, high blood pressure, dyslipidaemia, hyperinsulinemia, impaired fasting glucose, elevated serum triglycerides, low serum high-density lipoprotein cholesterol, abdominal obesity, and type 2 diabetes [1,2,3]. Furthermore, MetS is a strong indicator of an increased risk of cardiovascular morbidity and mortality [4]. It has been established that light-to-moderate alcohol consumption is associated with a reduced risk of cardiovascular mortality [5,6,7], while excessive drinking increases the risk of death, injury, violence, and various diseases, such as high blood pressure, heart diseases, stroke, liver diseases, mental health problems, and cancers of the breast, throat, oesophagus, liver, and colon [8,9]. However, the association between binge alcohol consumption and MetS is unclear.

In the Ellisras Longitudinal Study cohort, the prevalence of alcohol abuse ranged from 42.2% to 94% for participants aged 14 to 22 years [10]. The prevalence of MetS was 23.1% (8.6% males and 36.8% females) amongst Ellisras rural young adults (aged 18 to 30 years) [11]. However, the association between binge drinking and the components of MetS in this population has not yet been explored. The purpose of this study, therefore, was to investigate the association between binge alcohol consumption and the components of MetS amongst Ellisras rural young adults aged 21 to 31 years who were part of the Ellisras Longitudinal Study.

## 2. Method

### 2.1. Study Design and Sampling

The Ellisras Longitudinal Study (ELS) design and sampling methodology has been reported elsewhere [10,12]. A quantitative study applying a cross-sectional design was conducted. A total of 624 adults (306 males and 318 females), aged 21 to 31 years, who were part of the Ellisras Longitudinal Survey (ELS) participated in the current study. The Ethics Committee of the University of Limpopo granted ethical approval before the survey (ethical clearance number MREC/P/204/2013: IR), and the participants signed informed consent forms.

### 2.2. Anthropometry

All participants underwent a series of anthropometric measurements according to the International Society for the Advancement of Kinanthropometry [13]. Height was measured using a Martin anthropometer to the nearest 0.1 cm, with the head in the Frankfort plane and the subject being in an anatomical position. Body weight was measured without shoes and with light clothing to the nearest 0.1 kg on a calibrated electronic scale. Body mass index (BMI) was calculated by dividing weight by height in kilogram metres squared (kg/m^2^). Waist girth or circumference (WC) was measured with a flexible steel tape to the nearest 0.1 cm at the point midway between the lowest rib and the iliac crest.

### 2.3. Biochemical Parameters

Participants fasted for 8 to 10 h before blood collection. All blood collections were carried out in the morning in schools by registered nurses from Witpoort Hospital. Blood samples were then placed in a cooler box on ice (2–8 °C) on-site, before being transported to the laboratory at Witpoort Hospital situated in Ellisras rural area. Fasting blood samples were centrifuged at 2500 rpm for 15 min, before analysis, and stored in a bio-freezer at −80 °C for later analysis [11]. Briefly, fasting venous blood specimens were collected from all the participants for the measurement of fasting blood glucose (FBG), total cholesterol (TCHOL), triglycerides (TG), fasting insulin, low-density lipoprotein cholesterol (LDL-C), and high-density lipoprotein cholesterol (HDL-C). Blood specimens for the measurement of fasting plasma glucose (FPG) were drawn into fluoride tubes (Becton Dickinson, Plymouth, UK). The FPG was measured using the glucose oxidase method, on a Beckman LX20^®^ auto analyser (Beckman Coulter, Fullerton, CA, USA), after the samples were centrifuged within 4 h.

Diagnosis of the components of MetS used the new harmonised guidelines of the International Diabetes Federation (IDF), which requires a large WC (≥94 cm males and ≥80 cm females) plus two of the following criteria: reduced HDL-C (<1.0 mmol/L males; <1.3 mmol/L females), high TG (≥1.7 mmol/L), elevated BP (≥130/85 mm Hg), high FPG (≥5.6 mmol/L) [14], and high LDL-C (≥3.0 mmol/L) [15].

### 2.4. Screening for Alcohol Abuse

The CAGE questionnaire developed by Ewing [16] was used in the study. The CAGE acronym stands for cut-annoyed-guilty-eye and has four yes/no items constituting the screening test: (1) Have you ever felt that you ought to cut down on your drinking? (2) Have people annoyed you by criticising your drinking? (3) Have you ever felt bad or guilty about your drinking? (4) Have you ever had a drink first thing in the morning to steady your nerves or to get rid of a hangover (eye-opener)?

Binge drinkers were defined as individuals with a consumption pattern of five or more alcoholic beverages in the case of men (≥62.5 g), and four or more alcoholic beverages for women (≥50 g), on a single occasion at least once during the previous 30 days [17,18].

Current alcohol drinkers were defined as anyone who drank alcohol regularly for the past 30 days. The following questions were asked: “During the past 30 days, have you had at least one drink of any alcoholic beverage, such as beer, wine, a malt beverage, or liquor?”; “During the past 30 days, on the days when you drank, about how many drinks did you drink on average during the week and over the weekend?”; “During the past 30 days, what was the largest number of drinks you had on any occasion?” People who reported drinking in the past month were classified into three categories as less than four times in the past 30 days, more than two to three days a week in the past 30 days, and more than four days a week in the past 30 days.

Ever alcohol drinkers were those who answered that they tried to drink or they drank occasionally and had stopped. The question used was: “Have you ever drunk alcohol (at least once)?” Those who never drank alcohol, at the time of the survey, were considered non-drinkers. The onset (initiation) age for alcohol consumption was determined by the question: “If yes, indicate how old you were when you first tried drinking alcohol ____? How old were you when you first drank alcohol regularly ______?”

### 2.5. Statistical Analysis

All statistical analyses were performed using SPSS, Version 26 (SPSS Inc., Chicago, IL, USA). Shapiro–Wilk W test was used to test the normal distribution of the continuous variables, and descriptive statistics were run by gender. The Student *t*-test was used to test for significant differences between genders, while the Mann–Whitney test was used to test for significant differences between genders for variables that were not normally distributed. A chi-square test was used to compare nominal data, while Fisher’s exact test was used when the expected cell frequencies were small (less than five) [19]. Logistic regression analysis was applied to determine the association between alcohol abuse (binge drinking) and the components of MetS. Covariates associated with the outcome variable were included in the model (age, gender, and smoking). The statistical significance was set at *p* < 0.05.

## 3. Results

Table 1 presents the descriptive statistics of age and MetS components among Ellisras rural young adults aged 21 to 31 years. The data reported no differences in means between males and females (*p* = 0.257). Compared to males, females showed a significantly higher waist circumference (♀ 82.2 vs. ♂ 75.5 cm, *p* < 0.05) and mean insulin (♀ 6.7 vs. ♂ 4.6 mmol/L, *p* < 0.05). Males showed significantly high mean systolic blood pressure (♂ 125.9 mm Hg vs. ♀ 114.2 mm Hg, *p* < 0.05) and diastolic blood pressure compared to females (♂ 71.4 vs. ♀ 69.1 mm Hg, *p* < 0.05).

Table 2 presents the prevalence of various lifestyle characteristics and MetS components among Ellisras young adults aged 21 to 31 years. The overall prevalence of current smoking was 20.3%, higher among females (24.3%) compared with males (16. 2%, *p* = 0.012). The prevalence of current alcohol consumption (12.9%) and binge drinking (9%) were not significantly different across gender (*p*-value = 0.313 and 0.216, respectively). The prevalence of MetS components were as follows: abdominal obesity 28.8%, higher among females, 52.1% (*p* < 0.001); HDL cholesterol 54.8%, higher among females, 78.5% (*p* < 0.001); systolic blood pressure 20.3%, higher among males, 32.9% (*p* < 0.001); and triglycerides 9.7%, with no significant difference by gender (*p* = 0.075).

Table 3 shows the unadjusted associations between binge drinking, related factors, and the components of MetS among Ellisras young adults aged 21 to 31 years. The odds of abdominal obesity and MetS composite were significant (*p* < 0.001) among the older age group 25–31 (COR = 2.13, 95% CI = 1.37–3.34 and OR = 1.65, 95% CI = 1.12–2.41, respectively) compared to their younger counterparts (ages 21–24). All MetS components were significantly higher among males, except for triglycerides (OR = 1.21, 95% CI = 0.71–2.07). Peak alcohol consumption in one sitting during the weekdays (OR = 2.09, 95% CI = 1.00–4.50) and weekends (OR = 0.90, 95% CI = 1.32–11.47) during the past 30 days was significantly associated with cholesterol for more than 5 and 9 standard drinks for women and 5 to 15 standard drinks for men, respectively. Binge drinking was significantly (*p* < 0.05) associated with HDL (OR = 1.18, 95% CI = 1.02–5.29).

Table 4 shows the adjusted associations between binge drinking, related factors, and the components of MetS among Ellisras young adults aged 21 to 31 years. Abdominal obesity (OR = 0.03, 95% CI = 0.01–0.06), HDL-C (OR = 0.93, 95% CI = 0.62–1.13), hypertension (OR = 6.26, 95% CI = 3.35–11.45), and MetS (OR = 0.12, 95% CI = 0.08–0.18) were significantly associated (*p* < 0.05), and significantly lower in males for abdominal obesity, LDL-C, and MetS (*p* < 0.05). The older age group (25 to 31 years) was significantly associated with triglyceride (OR = 2.32, 95% CI = 1.06–5.11) and MetS (OR = 1.67, 95% CI = 1.08–2.58).

## 4. Discussion

This study aimed to investigate the association between binge alcohol consumption and the components of MetS amongst Ellisras rural young adults aged 21 to 31 years. Binge alcohol consumption was significantly associated with HDL-C, after adjusting for factors of binge drinking. The present results differed from the findings of Rosoff et al. [20], who reported a significant association between high-volume binge drinking and high cholesterol, triglycerides, and liver function enzyme levels. Furthermore, Klatsky and Gunderson [21] reported a significant association between binge drinking and hypertension, which was different from the findings of the current study. In the current study, diabetes was not associated with drinking less than four times in the past 30 days, and more than 5 to 9 standard drinks for women, and 5 to 15 standard drinks for men, during peak alcohol consumption in one sitting, during the past 30 days. Alcohol consists of a considerable amount of calories and sugar, thus binge drinking results in a build-up of fat and triglycerides in the blood, which might partially occlude the arteries and result in high blood pressure [17,18].

The current study acknowledges the varying definitions of binge alcohol consumption or risky drinking behaviour for comparison with related studies. Risky drinking behaviour was defined as drinking (Black Label, Castle, and Hansa) beer at a rate of five or more 750-mL bottles (less than 5.5% alcohol/volume) per day for males, and three or more 750-mL bottles (less than 5.5% alcohol/volume) per day for females [22,23]. Excessive alcohol consumption included heavy drinking (>15 drinks/week for men; >8 drinks/week for women), binge drinking (>5 drinks on an occasion for men; >4 drinks on an occasion for women) [17,18]. The National Institute on Alcohol Abuse and Alcoholism defined binge drinking as drinking >4 drinks per day for females and >5 drinks per day for males [24]. In the current Ellisras cohort, communal drinkers were more prevalent during weekends, for example, Fridays (4.9% for males and 7.3% for females) and Saturdays (6.1% for males and 7.7% for females), with females significantly higher on Fridays (*p* < 0.049) compared to males [10]. In the literature, it was found that men were more likely to consume alcohol consistently, since they presumably experienced more social problems. However, this differed with cultural, demographical, and historical characteristics [24,25].

Although moderate drinking of alcohol has positive effects on the heart [5,7], binge drinking has been shown to increase the cholesterol and triglyceride levels in the blood [26,27]. The increased build-up of fat in arterial walls progressively results in atherosclerosis, which may later develop into various cardiovascular diseases [28,29,30]. In the current study, the study participants were at increased risk of developing cardiovascular diseases because of their adverse drinking behaviour and MetS profile. This might be attributed to the fact that Ellisras is located around industrial mines and, hence, the income of most of the dwellers was relatively high, and this resulted in the increased drinking of alcohol.

The strength of the current study includes the baseline information collected on the same group of participants who were part of the ongoing Ellisras Longitudinal Study. The data collection method used in the ELS was standardised for alcohol consumption, as well as the components of MetS. The study has some limitations, despite these abovementioned strengths. The cross-sectional nature of the current study limits any temporal or causal relationship. The potential recall bias in the use of the alcohol questionnaire should not be ignored, even though this was partly addressed by adding specific time frames (weekends or during weekdays) as reference points.

## 5. Conclusions

Binge alcohol drinking was not significantly associated with MetS. However, a component of MetS (i.e., HDL-C) was significantly associated with binge drinking. Future studies should focus on the evidence needed to improve public health programs and policies aimed at reducing the prevalence of binge drinking.

## Figures and Tables

**Table 1:** Descriptive statistics of metabolic syndrome and the prevalence of binge alcohol consumption amongst Ellisras young adults aged 21 to 31 years.

Variable	Males *n* = 306		Females *n* = 318		*p*-Value
	Mean	(*SD*)	Mean	(*SD*)	
Age (years)	25.5	(1.92)	25.6	(2.07)	0.257
Fasting glucose (mmol/L)	5.5	(0.87)	5.6	(1.55)	0.097
Total cholesterol (mmol/L)	4.0	(0.92)	4.3	(1.11)	0.656
High-density lipoprotein (mmol/L)	1.2	(0.37)	1.1	(0.30)	0.001
Triglyceride * (mmol/L)	0.84	(0.63–1.18)	0.77	(0.62–1.17)	0.033
Low density lipoprotein (mmol/L)	2.6	(0.78)	2.9	(0.95)	0.001
Fasting insulin * (mmol/L)	4.6	(2.8–8.4)	6.5	(4.0–11.5)	0.001
Waist girth (cm)	75.1	(9.53)	82.1	(14.39)	0.0001
Systolic blood pressure (mm Hg)	125.9	(12.48)	114.2	(10.87)	0.001
Diastolic blood pressure (mm Hg)	71.4	(10.24)	69.1	(9.42)	0.003

* Median: 25 and 75 percentile; *SD*: standard deviation.

**Table 2:** Association between variables of binge drinking and variables of metabolic syndrome in Ellisras young adults aged 21 to 31 years.

Variable	Total *n* (%)	Female *n* (%)	Male *n* (%)	*p*-Value
**Age group (years)**				
Mean (*SD*)	25.6 (*SD* + 1.98)	25.6 (*SD* + 2.02)	25.4 (*SD* + 1.92)	
21–24	162 (29.7)	77 (27.6)	85 (31.8)	
25–31	384 (70.3)	202 (72.4)	182 (68.2)	
**Current smoker (total tobacco use)**				
No	490 (79.7)	240 (75.7)	254 (83.8)	
Yes	126 (20.3)	77 (24.3)	49 (16.2)	0.012
**Current alcohol consumption**				
No	541 (87.1)	272 (85.8)	269 (88.5)	
Yes	80 (12.9)	45 (14.2)	35 (11.5)	0.313
**Binge drinker**				
No	565 (91.0)	284 (89.6)	281 (92.4)	
Yes	56 (9.0)	33 (10.4)	23 (7.6)	0.216
**(1) Have you ever felt that you ought to cut down on your drinking?**				
Not applicable	527 (84.5)	263 (83.0)	264 (86.8)	
No	22 (3.5)	18 (5.7)	4 (1.3)	
Yes	72 (11.5)	36 (11.4)	36 (11.8)	0.988
**(2) Have people annoyed you by criticising your drinking?**				
Not applicable	527 (84.5)	263 (83)	264 (86.8)	
No	38 (6.1)	21 (6.6)	17 (5.6)	
Yes	56 (9.0)	33 (10.4)	23 (7.6)	0.199
**(3) Have you ever felt bad or guilty about your drinking?**				
Not applicable	527 (84.5)	263 (83.0)	264 (86.8)	
No	34 (5.4)	24 (7.6)	10 (3.3)	
Yes	60 (9.6)	30 (9.5)	30 (9.9)	0.989
**(4) Have you ever had a drink first thing in the morning to steady your nerves or to get rid of a hangover (eye-opener)**				
Not applicable	527 (84.5)	263 (83.0)	264 (86.8)	
No	14 (2.2)	9 (2.8)	5 (1.6)	
Yes	80 (12.8)	45 (14.2)	35 (11.5)	0.290
**Frequency of drinking in the past 30 days**				
Not applicable	541 (86.7)	272 (85.8)	269 (88.5)	
Less than four times in the past 30 days	37 (5.9)	19 (6.0)	18 (5.9)	0.899
More than two to three times a week in the past 30 days	43 (6.9)	26 (8.2)	17 (5.6)	0.198
**Peak alcohol consumption in one sitting during weekdays in the past 30 days**				
Not applicable	540 (86.5)	271 (85.5)	269 (88.5)	
Less than 4 drinks per sitting for both men and women	46 (7.4)	28 (8.8)	18 (5.9)	0.164
More than 5 to 9 standard drinks for women, and 5 to 15 standard drinks for men	35 (5.6)	18 (5.7)	17 (5.6)	0.895
**Peak alcohol consumption in one sitting over weekends in the past 30 days**				
Not applicable	565 (90.5)	287 (90.5)	278 (91.4)	
Less than 4 drinks per sitting for both men and women	14 (2.2)	7 (2.2)	7 (2.3)	0.984
More than 5 to 9 standard drinks for women, and 5 to 15 standard drinks for men	42 (6.7)	23 (7.3)	19 (6.3)	0.575
**Onset age of drinking**				
Not applicable	541 (86.7)	272 (85.8)	269 (88.5)	
Below 15 years	23 (3.7)	8 (2.5)	15 (4.9)	0.145
Equal to and above 15 years	57 (9.1)	37 (11.7)	20 (6.6)	0.145
**Components of metabolic syndrome by gender**				
**Waist girth (abdominal obesity)**				
Normal	442 (71.2)	152 (47.9)	290 (95.4)	
High	179 (28.8)	165 (52.1)	14 (4.6)	<0.001
**Total cholesterol**				
Normal	51 (82.9)	247 (77.9)	270 (88.8)	
High	104 (16.7)	70 (22.1)	34 (11.2)	<0.001
**Blood glucose**				
Normal	337 (54.3)	166 (52.4)	171 (56.3)	
High	284 (45.7)	151 (47.6)	133 (43.8)	<0.001
**HDL cholesterol**				
Normal	281 (45.2)	68 (21.5)	213 (70.1)	
High	340 (54.8)	249 (78.5)	91 (29.3)	<0.001
**LDL cholesterol**				
Normal	403 (64.9)	181 (57.1)	222 (73.0)	
High	218 (35.1)	136 (42.9)	82 (27.0)	<0.001
**Triglycerides**				
Normal	561 (90.3)	289 (91.2)	272 (89.5)	
High	60 (9.7)	28 (8.8)	32 (10.5)	0.075
**Systolic blood pressure**				
Normal	495 (79.7)	291 (91.8)	204 (67.1)	
High	126 (20.3)	26 (8.2)	100 (32.9)	<0.001
**Diastolic blood pressure**				
Normal	578 (93.1)	301 (95)	277 (91.1)	
High	43 (6.9)	16 (5.0)	27 (8.9)	0.06
**Hypertension (high blood pressure)**				
Normal	537 (86.5)	301 (95)	236 (77.6)	
High	84 (13.5)	16 (5.0)	68 (22.4)	<0.001
**MetS composite**				
Normal	351 (56.5)	113 (35.6)	238 (78.3)	

HDL = high-density lipoprotein; LDL = low-density lipoprotein; MetS = metabolic syndrome.

**Table 3:** Unadjusted associations between binge drinking, related factors, and components of metabolic syndrome among Ellisras young adults aged 21 to 31 years.

Predictor Variables	Waist Girth	Diabetes	HDL-C	Total Cholesterol	Hypertension	Triglycerides	MetS Composite
**Gender**							
Female (Ref)							
Male	0.04 (0.02–0.08) ***	0.85 (0.62–1.17)	0.11 (0.08–0.16) ***	0.44 (0.28–0.69) ***	5.42 (3.06–9.59) ***	1.21 (0.71–2.07)	0.15 (0.10–0.21) ***
**Age groups (years)**							
21–24 (Ref)							
25–31	2.13 (1.37–3.34) **	0.95 (0.66–1.38)	1.12 (0.77–1.62)	1.36 (0.85–2.19)	1.33 (0.76–2.33)	2.30 (1.05–5.02) *	1.65 (1.12–2.41) *
**Current smoker**							
Yes	1.30 (0.88–1.98)	1.16 (0.78–1.72)	0.91 (0.62–1.36)		1.08 (0.61–1.89)	0.76 (0.37–155)	1.04 (0.70–1.55)
**Binge drinker**							
Yes	0.99 (0.59–1.67)	0.91 (0.56–1.46)	1.35 (0.84–2.19)	1.41 (0.79–2.52)	1.57 (0.85–2.92)	1.40 (0.68–2.89)	1.01 (0.63–1.62)
**Frequency of drinking in the past 30 days**							
Less than four #	0.79 (0.36–1.69)	1.60 (0.82–3.13)	1.38 (0.70–2.74)	1.17 (0.5–0 2.74)	1.84 (0.81–4.18)	1.50 (0.56–4.02)	1.25 (0.64–2.43)
More than and equal to four #	1.24 (0.42–3.68)	0.42 (0.13–1.34)	0.72 (0.26–200)	1.84 (0.57–5.89)	1.62 (0.45–5.87)	0.66 (0.09–5.13)	0.32 (0.09–1.14) *
**Peak alcohol consumption in one sitting during weekdays in the past 30 days**							
Less $	0.67 (0.32–1.38)	0.91 (0.51–1.66)	1.08 (0.59–1.98)	1.05 (0.48–2.32)	1.38 (0.62–3.08)	1.15 (0.44–3.04)	0.82 (0.45–1.52)
More $	1.70 (0.85–3.43)	1.0 (0.50–1.98)	1.08 (0.59–1.98)	2.09 (1.00–4.50) *	1.65 (0.70–3.91)	1.61 (0.60–4.32)	1.40 (0.45–1.52)
**Peak alcohol consumption in one sitting over weekends in the past 30 days**							
Less $	1.38 (0.46–4.18)	2.17 (0.72–6.56)	0.61 (0.21–2–1.79)	3.90 (1.32–11.47) **	0.49 (0.06–3.76)	1.58 (0.35–7.22)	0.97 (0.33–2.84)
More $	1.12 (0.57–2.40)	0.57 (0.30–1.11) *	2.17 (1.09–4.32) *	0.81 (0.34–1.99)	1.83 (0.84–3.98)	2.00 (0.84–4.69)	1.20 (0.64–2.24)
**Binge drinking**							
Yes	1.54 (0.87–2.74)	0.81 (0.46–1.41)	1.18 (1.02–3.29) *	0.78 (0.43–1.43)	1.25 (0.58–2.65)	1.64 (0.73–3.66)	1.44 (0.83–2.50)

* *p* < 0.05, ** *p* < 0.01, *** *p* < 0.001, # Less than four times in the past 30 days, # More than two to three times a week in the past 30 days, $ Less than 4 standard drinks per sitting for both men and women, $ More than 5 to 9 standard drinks for women, and 5 to 15 standard drinks for men.

**Table 4:** Adjusted associations between binge drinking, related factors, and components of metabolic syndrome among Ellisras young adults aged 21 to 31 years.

Predictor Variables	Waist Girth	Diabetes	HDL-C	LDL-C	Hypertension	Triglycerides	MetS Composite
**Sex**							
Female (Ref)							
Male	0.03 (0.01–0.06) *	0.83 (0.59–1.17)	0.93 (0.62–1.13) ***	0.45 (0.31–0.66) ***	6.20 (3.35–11.45) ***	1.22 (0.67–2.22)	0.12 (0.08–0.18) ***
**Age groups (years)**							
21–24 (Ref)							
25–31	1.07 (0.73–1.54) **	1.07 (0.77–1.55)	0.97 (0.62–1.50)	1.75 (1.16–2.65) *	1.45 (0.81–2.58)	2.32 (1.06–5.11) *	1.67 (1.08–2.58) *
**Current smoker**							
Yes	1.07 (0.62–1.83)	1.14 (0.75–1.75)	1.34 (0.81–2.21)	1.21 (0.77–1.91)	0.76 (0.41–1.41)	1.43 (0.64–3.17)	1.24 (0.77–2.00)
**Binge drinker**							
Yes	1.14 (0.54–2.40)	0.73 (0.39–1.34)	2.64 (1.23–5.65) *	0.55 (0.22–1.06)	1.29 (0.55–3.04)	1.36 (0.54–3.43)	1.33 (0.68–2.60)

* *p* < 0.05, ** *p* < 0.01, *** *p* < 0.001.
